# The Diagnostic Value of Fluoro-18 Fluorodeoxyglucose (F-18 FDG) PET/CT in Fever or Inflammation of Unknown Origin: A Retrospective Study at a Rheumatology Clinic

**DOI:** 10.7759/cureus.24192

**Published:** 2022-04-16

**Authors:** Tahir Saygın Öğüt, Funda Erbasan, Mustafa Ender Terzioğlu, Gokhan Tazegul, Veli Yazısız

**Affiliations:** 1 Rheumatology, Akdeniz University, Antalya, TUR; 2 Internal Medicine, Ankara Polatlı Duatepe State Hospital, Ankara, TUR

**Keywords:** inflammation, fluorodeoxyglucose, positron emission tomography, inflammation of unknown origin, fever of unknown origin

## Abstract

Introduction

Further diagnostic procedures are necessary for patients with fever of unknown origin (FUO) and unknown cause of inflammation (inflammation of unknown origin - IUO) for the identification of the definitive diagnosis. The aim of this study was to evaluate the contribution and roles of F-18 FDG PET/CT (fluoro-18 fluorodeoxyglucose-positron emission tomography/computed tomography) in the diagnostic process of patients with FUO/IUO.

Methods

The data of 58 patients who had F-18 FDG PET/CT scans for FUO/IUO were re-evaluated retrospectively. The relationships between definitive diagnosis and fluorodeoxyglucose uptake and SUVmax (maximum standardized uptake value) were examined.

Results

Rheumatic disease was diagnosed in 26 patients (44.5%), malignancy in 20 patients (34.5%), and infectious diseases in six patients (10.3%). The most prevalent rheumatic disease in patients with FUO/IUO was systemic vasculitis (n:10, 17.2%), especially large vessel vasculitis. There were 37 patients (63.7%) with clinically significant true positive fluorodeoxyglucose uptake. True positive fluorodeoxyglucose uptake was significantly higher in patients diagnosed with malignancy (85%, 17/20 patients) compared to other diagnoses. Fluorodeoxyglucose uptake above physiological levels was determined in 15 of the 26 patients (57.6%) diagnosed with rheumatic diseases.

Conclusion

The results of this study showed that F-18 FDG PET/CT is a useful imaging modality in FUO/IUO patients, who present a challenging diagnostic process for clinicians. In addition to malignancies, the presence of chronic inflammatory diseases, especially early period systemic vasculitis, were diagnosed in these patients.

## Introduction

Fever of unknown origin (FUO) and inflammation of unknown origin (IUO) are conditions with difficult diagnostic processes for clinicians. FUO was first described by Petersdorf and Beeson and is defined as a fever of ≥38.3°C, lasting at least 3 weeks, for which no cause can be established despite hospitalisation for 1 week [1]. There are several diseases in the etiology of FUO and when grouped according to literature these have been determined as infection (21-54%), neoplasms (6-31%), non-infectious inflammatory diseases (13-24%), and other diseases (4-6.5%) [2].

Inflammation of unknown origin (IUO) is accepted as elevated prolonged inflammatory markers and temperature <38.3°C [3]. Although the clinical presentations of FUO and IUO are different from each other, the diseases in the etiologies are similar [4].

In the process of investigating the etiology of FUO and IUO, many tests are applied to patients and they are exposed to various treatments. Medical history, physical examination, laboratory tests, peripheral blood and urine cultures, standard radiography, echocardiography, and abdominal ultrasonography are some of these tests. According to some studies, in 30-50% of patients with FUO/IUO, a definitive diagnosis cannot be made [3].

In patients with malignancy, F-18 FDG PET/CT (flouro-18 fluorodeoxyglucose positron emission tomography/computed tomography) is a body imaging method that is useful in disease grading and determining the localisation of metastatic foci [5]. F-18 FDG PET/CT may be helpful in diagnosing the underlying disease in patients with FUO/IUO [4,6]. In addition to oncological diseases, F-18 FDG PET/CT seems to be a method that can be used in inflammatory diseases where glucose uptake is increased. There are reports in the literature that it is effective in chronic inflammatory diseases such as systemic vasculitis and sarcoidosis, and could be used as a diagnostic and treatment method in cases of these diseases [6]. In recent years, F-18 FDG PET/CT has started to be used more often to discount focal infection and malignancy as a cause of fever.

The aim of this study was to evaluate the contribution of F-18 FDG PET/CT imaging to diagnosis in patients tested because of FUO and IUO, and to define the regions of involvement and patterns seen on F-18 FDG PET/CT in inflammatory diseases.

## Materials and methods

Approval for this study was granted by the Ethics Committee of Akdeniz University Medical Faculty (Decision no: 2018/259) and all the procedures were applied in compliance with the Declaration of Helsinki. Permission for the use of the data was obtained from the hospital management.

A retrospective evaluation was made of the data of patients aged >18 years who were followed up in the Rheumatology Clinic of Akdeniz University Medical Faculty Hospital between January 2014 and December 2017. The F-18 FDG PET/CT images and the data related to routine laboratory test results, pre-diagnoses, definitive diagnoses, and the treatments applied were retrieved from the hospital information system records.

Clinical evaluation

The demographic, clinical, and laboratory data of the patients were evaluated retrospectively. Using the hospital information processing system, the anamneses, symptoms, findings, and results of the laboratory tests and imaging methods were examined.

For the patients hospitalised because of FUO/IUO, the data were retrieved from the records of the standard tests of full blood count, erythrocyte sedimentation rate (ESR), C-reactive protein (CRP), procalcitonin, routine biochemistry tests, full urine analysis, peripheral smear, Wright tube agglutination test, Rose Bengal test, ferritin, hepatitis B, hepatitis C, and HIV serology, thyroid function tests, purified protein derivative (PPD) test, culture results (blood, urine, throat, mucus, and faeces cultures), autoantibody tests [rheumatoid factor (RF), antinuclear antibody (ANA), extractable nuclear antigen (ENA) antibodies, antineutrophil cytoplasmic antibody (ANCA)**]**, posteroanterior pulmonary radiograph, abdominal ultrasonography, echocardiography, and the tests applied as necessary of monospot test, Epstein-Barr virus profile [Epstein-Barr virus nuclear antigen (EBNA), viral-capsid antigen (VCA)]​​​​​, neck, thorax, and abdomen CT, magnetic resonance imaging (MRI), and bone marrow aspiration and biopsy. The patients were grouped according to the final diagnoses, as those with etiology of infectious causes, inflammatory pathologies, malignancies, and those who could not be diagnosed.

F-18 FDG PET/CT imaging and evaluation

The electronic records and reports of the F-18 FDG PET/CT images of the patients were obtained from the hospital information processing system and evaluated. The images had been taken as 16-slice multidetector CT integrated with a high-resolution Siemens Biograph TruePoint 16 PET/CT with 3D mode (Siemens, Erlangen, Germany). The maximum standard uptake value (SUVmax) of F-18 FDG (fluoro-18 fluorodeoxyglucose) in the areas of involvement was calculated by a Nuclear Medicine specialist using the formula:

SUV = activity concentration in the tissue (mCi/ml or Bq/ml)/injected activity (mCi or Bq)/body dimensions*

*body weight, body surface area or non-fat body mass can be used. The most frequently used is body weight, in which case the final unit is kg/ml.

Definitive diagnoses were made according to the clinical, laboratory, imaging, and histopathological evaluations. The decision of whether or not the F-18 FDG PET/CT had helped in reaching the final diagnosis was made by the research team. F-18 FDG PET/CT was evaluated from a diagnostic perspective in all the patients with FUO/IUO applied with F-18 FDG PET/CT and according to the final diagnosis categories.

Statistical analysis

Data obtained in the study were analyzed statistically using SPSS Statistics for Windows version 23.0 software (IBM Corp., Armonk, USA). Continuous variables were stated as mean ± standard deviation values and categorical data as number (n) and percentage (%). In the statistical analyses, the Chi-square test was used for categorical data, the Student’s t-test for numerical data with normal distribution, and the Mann-Whitney U-test for data not showing normal distribution. A value of p<0.05 was accepted as statistically significant in all the statistical tests. Categorisation was applied according to whether or not F-18 FDG PET/CT contributed to the final diagnosis. The sensitivity, specificity, accuracy, positive predictive value (PPV) and negative predictive value (NPV) were calculated for these patient groups using the MedCalc diagnostic test calculator (MedCalc Software Ltd., Ostend, Belgium) [7].

## Results

In the defined study period, F-18 FDG PET/CT images were taken of a total of 286 patients, of which the indication for imaging was FUO and/or IUO in 58 patients. The general characteristics of the patients are shown in Table 1.

**Table 1:** General characteristics of the patients included in the study IUO: Inflammation of unknown origin, FUO: Fever of unknown origin, SD: Standard deviation, CRP: C-reactive protein, ESR: erythrocyte sedimentation rate, LDH: lactate dehydrogenase.

	IUO (n:33)	FUO (n:25)
Gender (Male)	15 (45.5%)	15 (60.0%)
Age (years; mean ± SD)	60,8±11.8	45.3±17
CRP (mg/dl; mean ± SD)	6.8±6.4	10.8±7.2
ESR (mm/hour; mean ± SD)	76.6±22.7	57±29.9
Hemoglobin (g/dl, mean ± SD)	9.4±1.5	10±1.7
LDH (U/L; mean ± SD)	233±167	398±378
Leukocytes (10^3^/mm^3^; mean ± SD)	9.1±4.7	10.3±6.2
Platelet (10^3^/mm3; mean ± SD)	346±165	314±143

In a total of 58 patients, a diagnosis was made of rheumatological disease in 26 (44.5%), malignancy in 20 (34.5%), and the etiology was associated with infectious causes in six (10.3%). In six (10.3%) of the F-18 FDG PET/CTs taken, there was no diagnosis, it was unknown, or reported as not diagnostic. Of the patients with IUO, the final diagnosis of the F-18 FDG PET/CTs was reported as rheumatological disease in 15 (45.4%) patients, malignancy in 12 (36.4%), infectious causes in three (9.1%), and no diagnosis/unknown/not diagnostic in three (9.1%). In the patients with FUO, the final diagnosis of the F-18 FDG PET/CTs was determined to be rheumatological disease in 11 (44%) patients, malignancy in eight (32%), infectious causes in three (12%), and no diagnosis/unknown/not diagnostic in three (12%). The mean SUVmax value was determined to be 5.1±4.0 in patients diagnosed with rheumatological disease and 13.3±7.9 in patients diagnosed with malignancy. The SUVmax values were determined to be statistically significantly higher in the patients diagnosed with malignancy (p<0.001) (Table 2).

**Table 2:** Reasons for requesting F-18 FDG PET/CT and definitive diagnoses IUO: Inflammation of unknown origin, FUO: Fever of unknown origin, SUV: Standart uptake volume, SD: Standart deviation, ANCA: Anti-neutrophil cytoplasmic antibodies, F-18 FDG PET/CT: fluoro-18 fluorodeoxyglucose-positron emission tomography/computed tomography * The numbers in the parentheses denote the number of cases for each defined diagnosis.

	IUO		FUO		Total		SUVmax (Mean±SD)	Clinical Diagnosis*
	n %		n %		n %			
Inflammatory rheumatic diseases	15	45.4	11	44.0	26	44.5	5.1±4.0*	Adult Still Disease (7), Takayasu arteritis (5), seronegative spondyloarthropathy (3), polymyalgia rheumatica (2), ANCA-related vasculitis (2), temporal arteritis (2), connective tissue diseases (1), panniculitis (1), Kawasaki disease (1), sarcoidosis (1), pemphigus vulgaris (1).
Malignancies	12	36.4	8	32.0	20	34.5	13.3±7.9*	Lymphoma (5), colon adenocarcinoma (3), multiple myeloma (3), non-small-cell lung cancer (3), myelodisplastic syndrome (1), stomach cancer (1), leukemia (1), Kaposi sarcoma (1), sarcoma (1), primary unknown adenocarcinoma (1).
Infections	3	9.1	3	12.0	6	10.3		Pneumonia (2), osteomyelitis (1), infective endocarditis (1), leishmania (1), tuberculous lymphadenitis (1).
Non-diagnostic	3	9.1	3	12.0	6	10.3		-

Of the total 58 patients with F-18 FDG PET/CT taken because of FUO/IUO, vasculitis was diagnosed in 10 (17.2%), large vessel vasculitis in seven (12%) (five Takayasu arteritis, two temporal arteritis), and other forms of vasculitis in three (two anti-neutrophil cytoplasmic antibodies [ANCA]-related vasculitis, one Kawasaki disease).

High F-18 FDG PET/CT uptake was determined in 47 of the 58 patients. No relationship was determined between the increase in F-18 FDG uptake and final diagnosis in 10 patients. The F-18 FDG PET/CT imaging was determined to contribute to diagnosis in 37 (63.7%) patients. Significant F-18 FDG uptake was determined in 17/20 (85.0%) patients diagnosed with malignancy, and in 15/26 (57.6%) patients diagnosed with rheumatological disease. From these findings, The F-18 FDG PET/CT in patients followed up because of FUO/IUO was determined to have 88.1% sensitivity (95% CI: 74-96%), 37.5% specificity (95% CI: 15-64%), and 74.14% diagnostic accuracy (95% CI: 60-84%). The sensitivity, specificity, diagnostic accuracy, positive predictive, and negative predictive values are shown in Table 3.

**Table 3:** Evaluations of F-18 FDG PET/CT from a diagnostic perspective according to the final diagnosis categories in all the patients with FUO/IUO PET/CT: Positron emission tomography/computed tomography, PPV: Positive predictive value, NPV: Negative predictive value, FUO: Fever of unknown origin, IUO: Inflammation of unknown origin

Category	Contribution to final diagnosis	PET/CT positive	PET/CT negative	Sensitivity	Specificity	Accuracy	PPV	NPV
All patients (n:58)	Yes	37/47 (78.7%)	5/11 (45.4%)	88.1	37.5	74.1	78.7	54.6
	No	10/47 (21.2%)	6/11 (54.5%)					
Rheumatological disease (n:26)	Yes	15/21 (71.4%)	4 (80%)	78.9	14,3	61.5	71.4	20.0
	No	6/21 (28.6%)	1 (20%)					
Malignancy (n: 20)	Yes	17 (94.4%)	1 (50%)	94.4	50.0	90.0	94.4	50.0
	No	1 (5.6%)	1 (50%)					
Infectious disease (n:6)	Yes	5 (83.3%)	0	--	--	--	--	--
	No	1 (16.7%)	0					

Of the cases where increased F-18 FDG uptake contributed to the final diagnosis, chronic inflammatory rheumatismal disease was determined in 15, malignancy in 17, and infectious disease in five. The F-18 FDG PET/CT result was accepted as contributing to the diagnosis in 17 patients diagnosed with malignancy. Of the 15 patients diagnosed with rheumatological disease and the F-18 FDG PET/CT result was evaluated as contributing to the diagnosis, large vessel vasculitis was diagnosed in six (five Takayasu arteritis, one temporal arteritis) and Kawasaki disease in one. In the 10 patients where involvement was evaluated as not contributing to the diagnosis, the final diagnosis was vasculitis in two patients, polymyalgia rheumatica in one, colon adenocarcinoma in one, pemphigus vulgaris in one, adult Still disease in one, infective endocarditis in one, and in two patients it was accepted as no diagnosis/unknown/not diagnostic.

F-18 FDG uptake was not determined on a pathological surface of any organ in 11 patients. Inflammatory disease was diagnosed in five of these patients (two adult Still disease, one temporal arteritis, one seronegative spondyloarthropathy, one panniculitis), multiple myeloma in one, and myelodysplastic syndrome in one. In four patients, a definitive diagnosis could not be made.

The comparisons of organ involvement on the F-18 FDG PET/CT scans of patients taken because of FUO/IUO and diagnosed with malignancy or rheumatological disease are shown in Table 4. Lymph node involvement was observed in 13/20 (65%) patients diagnosed with malignancy and in 7/26 (27%) patients diagnosed with rheumatological disease. Lymph node involvement was found to be statistically significantly higher in the malignancy group (p=0.013). Bone involvement was observed in 6/20 (30%) patients diagnosed with malignancy and in none of the patients in the rheumatological disease group. F-18 FDG uptake in bones was determined to be statistically significantly higher in the patients diagnosed with malignancy (p=0.005). Vascular F-18 FDG uptake was determined in 7/26 patients in the rheumatological disease group and in 1/20 (5%) of the malignancy group (p=0.001). No significant difference was determined between the groups in respect of lung, bone marrow, liver, spleen, and joint involvement (Table 4).

**Table 4:** Comparisons of organ involvement on F-18 FDG PET/CT taken because of FUO/IUO in the patients diagnosed with malignancy or rheumatological disease *Chi-square test FUO: Fever of unknown origin, IUO inflammation of unknown origin, F-18 FDG PET/CT: fluoro-18 fluorodeoxyglucose-positron emission tomography/computed tomography

	Malignancy	Inflammatory Disease	p*
	(n:20)	(n=26)	
Lymph Node	13 (65%)	7 (27.0%)	0.013
Bone marrow	3 (15%)	5 (19.2%)	0.487
Lungs	6 (30%)	5 (19.2%)	0.438
Liver	2 (10%)	0 (0%)	0.192
Spleen	3 (15%)	2 (7.7%)	0.392
Bones	6 (30%)	0 (0%)	0.005
Vascular	1 (5%)	7 (26.9%)	0.001
Joints	0 (0%)	2 (7.7%)	0.303

## Discussion

FUO or IUO are conditions that make it difficult for clinicians to make a definitive diagnosis. They may originate from up to potentially 200 different diseases and the majority of patients have non-specific symptoms and few clues. F-18 FDG PET/CT is superior to conventional imaging techniques as it allows the imaging of a wider area. F-18 FDG is a marker of increased cell metabolism and is therefore taken up not only by malignant cells but also by cells related to infectious and inflammatory processes [8,9]. In this retrospective study, the contribution of F-18 FDG PET/CT to the diagnostic process was investigated in patients followed up because of FUO/IUO who were applied with F-18 FDG PET/CT imaging.

A definitive diagnosis was made in 52 (89.7%) of the 58 patients applied with F-18 FDG PET/CT imaging to investigate the etiology of FUO/IUO. In 37 of these patients, the diagnosis was provided by F-18 FDG PET/CT. In literature, the rates of cases for which diagnosis could not be made vary from 9% to 50% for FUO (91-100) and from 11% to 60% for IUO [4]. In the current study, the diagnosis could not be made for six patients, and F-18 FDG PET/CT did not contribute to the diagnostic process in 16 patients.

Of the patients applied with F-18 FDG PET/CT because of FUO/IUO, inflammatory rheumatological disease was diagnosed in 44.5%, malignancy in 34.5%, and infectious disease in 10.3%. There have recently been many studies in the literature about the diagnostic role of F-18 FDG PET/CT in FUO/IUO [4,6,8-13].

The diagnostic contribution of F-18 FDG PET/CT in patients diagnosed with malignancy (85%) was determined to be higher than for patients diagnosed with rheumatological disease (57.6%). The diagnostic contribution in patients examined because of FUO/IUO was found to be 63.7%. F-18 FDG PET/CT in patients followed up because of FUO/IUO was determined to have 88.1% sensitivity (95% CI: 74-96%), 37.5% specificity (95% CI: 15-64%), and 74.14% diagnostic accuracy (95% CI: 60-84%). The results of a newly published study showed a sensitivity of 80.2% and specificity of 89.8% for F-18 FDG PET/CT in contributing to the final diagnosis [14]. 

In a prospective study by Schönau et al, 240 patients with F-18 FDG PET/CT scans taken because of FUO/IUO were evaluated. In IUO, rheumatological diseases (62%) and infectious diseases (11.3%) were determined, and in FUO, rheumatological disease (47.2%) and infectious diseases (15.3%). The rate of diagnosis was determined as 71.6% and the contribution of F-18 FDG PET/CT to the diagnosis (true positive) was 56.7% [8]. In another study of 317 IUO patients, the F-18 FDG PET/CT results were classified as true positive in 49.8% of patients and contributory in 75.1% of overall IUO patients [15]. In the current study, the rate of true positivity of F-18 FDG PET/CT was 63.7%, but this rate was determined as 10.3% in infectious diseases.

A retrospective study of 50 FUO patients in Greece reported the diagnosis rate to be 78% overall and 40% in infectious diseases. When the data of these two studies from geographically close countries are evaluated, the difference in the percentage of infectious diseases is striking. The reason for this could be that the current study did not include patients from the Infectious Diseases Clinic, and that the patients classified as no diagnosis/unknown/not diagnostic could have had an etiology of infectious disease. Another reason could have been that the current study was conducted in the western Mediterranean region where few cases of tuberculosis are seen because of the temperate climate [16].

In a study in China in 2018, Wang et al determined infectious and rheumatological diseases at the rates of 33% and 32.4%, respectively in FUO/IUO patients, and the diagnosis was determined to be made at the rate of 84.5% [17]. The rate of 89.7% of diagnoses made in the current study was seen to be similar to the results of other recent studies. However, while the rate of rheumatological diseases increases over time in FUO/IUO patients, the rate of infectious causes decreases.

It has been reported that F-18 FDG PET/CT provides important clinical information in respect of the underlying pathological condition in 42-92% of FUO/IUO patients [12]. The contribution of FDG involvement on F-18 FDG PET/CT of patients followed up because of FUO/IUO was determined as 56.7% in a prospective study by Schönau et al [8], as 72% by Georga et al [16], and as 77.4% by Wang et al [17]. In the current study, a definitive diagnosis was made in 89.7% of the FUO/IUO patients applied with F-18 FDG PET/CT imaging and the F-18 FDG PET/CT was seen to provide additional information in 63.7% of the patients. These findings demonstrate that F-18 FDG PET/CT is of benefit in these patient groups which have diagnostic challenges. However, as this is a high-cost imaging method, there is a need for cost-benefit analyses. It has been stated that F-18 FDG PET/CT performed early according to the patient characteristics and etiological clues could be helpful in shortening the length of stay in hospital and limiting the medical costs [18].

The most common cause of FUO/IUO in the current study cohort was inflammatory rheumatological diseases, similar to the findings of studies from developed countries. The most common diagnosis in the inflammatory rheumatological disease group was large vessel vasculitis (five Takayasu arteritis, two temporal artertis). The high diagnostic success of F-18 FDG PET/CT in active large vasculitis has been shown in previous studies [19,20]. In all the current study patients diagnosed with Takayasu arteritis, there was significant vascular F-18 FDG involvement on F-18 FDG PET/CT. Temporal arteritis was diagnosed in two patients, of which there was significant FDG involvement in one. It has been reported in the literature that F-18 FDG PET/CT is a good option for the imaging of vascular wall lesions, especially in patients with large-vessel vasculitis who present with atypical findings [21]. In respect of diagnosis, F-18 FDG PET/CT has been determined to have 76% sensitivity and 93% specificity in large vessel vasculitis and 83% sensitivity and 90% specificity in giant-cell arteritis [21]. In a study by Soussan et al, F-18 FDG PET/CT was found to have 87% sensitivity and 73% specificity in Takayasu arteritis [22]. In the current study, significant FDG involvement was seen in 6/7 (85%) patients diagnosed with large vessel vasculitis. These findings support the view that F-18 FDG PET/CT imaging could be a useful imaging method in the determination of large vessel vasculitis. However, there is still uncertainty about the use of F-18 FDG PET/CT in the follow-up of patients with large artery vasculitis. There are conflicting data in respect of the correlation between FDG involvement and disease activity interpreted clinically, biologically, and on MRI [23].

In 3/5 of the current study patients diagnosed with Takayasu arteritis, there were no findings suggestive of Takayasu arteritis in other imaging methods performed before F-18 FDG PET/CT, and the diagnosis of aortitis (early-stage Takayasu arteritis) was made based only on the F-18 FDG PET/CT imaging. In 4/5 patients, no pathological findings were determined on Doppler ultrasonography, and vessel wall abnormalities were only seen on F-18 FDG PET/CT. In this study, sensitivity and specificity analysis of F-18 FDG PET/CT in the imaging of aortitis was not performed in a large Takayasu cohort, but it was shown that aortitis could be only be imaged with F-18 FDG PET/CT in some patients who did not have the classic findings of Takayasu arteritis in the physical examination and other imaging methods. It is known that the diagnosis of Takayasu arteritis may be made months or even years after the onset of the first symptoms [24]. Suspicion of disease is raised with murmur, pulse alterations and ischaemic symptoms which emerge after strictures have developed in vessel walls and diagnosis can be made. Old diagnostic criteria [25] are far removed from the diagnosis of patients in the early period, as most of the criteria indicate the presence of established vessel lesions [26,27]. Stricture and occlusion in the lumen of large vessels in Takayasu arteritis are complications of even late-term lesions of the disease [28]. All the events of the early-stage disease occur in the adventitia and media layer of large vessels. At this stage there is an increase in cytokines such as interleukin (IL)-6 and tumour necrosis factor (TNF)-alpha because of an increased acute phase and chronic inflammation, and additional problems emerge such as subfebrile fever, aches, fatigue, and chronic disease anemia. However, as there is no narrowing in the lumen at this stage, blood flow continues as normal and the physical examination findings are found to be completely normal. There are difficulties in the diagnosis of patients in this period and the findings of the current study show that F-18 FDG PET/CT could be a good alternative diagnostic method. Diagnosis of these patients in the early stage of Takayasu arteritis allows the opportunity for early treatment and will possibly provide advantages in preventing the development of permanent vascular pathologies in the chronic stage. The current study results reveal the necessity for performing F-18 FDG PET/CT imaging in patients with FUO/IUO to avoid overlooking potential early-stage Takayasu arteritis.

In a study by Arnow and Flaherty, temporal arteritis was determined in the etiology of 15% of FUO patients [29]. In the current study, temporal arteritis was determined in two (3.2%) of the 62 patients applied with F-18 FDG PET/CT because of FUO/IUO. This difference in the rates could be attributed to the fact that temporal arteritis is seen more often in a North American population or that temporal arteritis can be diagnosed more often without the application of F-18 FDG PET/CT.

This study had some strong aspects and some limitations. The strong aspects can be said to be that it was conducted in a single centre, it was possible to reach all the patients applied with F-18 FDG-PET/CT, monitoring of the patients after F-18 FDG PET/CT was performed in the same clinic, and the long-term results of the patients were available. The most important limitations of the study were that it was retrospective in design and the F-18 FDG PET/CT scans were evaluated by different specialists. In addition, as FUO/IUO patients who were not applied with F-18 FDG PET/CT were not included in the study, so no comparisons with this group could be made.

## Conclusions

The results of this study demonstrate that F-18 FDG PET/CT is a useful imaging method in FUO/IUO patients for whom there are difficulties in the diagnostic process. In addition to malignancies in these patients, inflammatory disease, and especially systemic vasculitis can be diagnosed.

## References

[1] Petersdorf RG, Beeson PB (1961). Fever of unexplained origin: report on 100 cases. Medicine.

[2] Durack DT, Street AC (1991). Fever of unknown origin--reexamined and redefined. Curr Clin Top Infect Dis.

[3] de Kleijn EM, Knockaert DC, van der Meer JW (2000). Fever of unknown origin: a new definition and proposal for diagnostic work-up. Eur J Intern Med.

[4] Vanderschueren S, Del Biondo E, Ruttens D, Van Boxelaer I, Wauters E, Knockaert DD (2009). Inflammation of unknown origin versus fever of unknown origin: two of a kind. Eur J Intern Med.

[5] Kim TY, Kim WB, Ryu JS, Gong G, Hong SJ, Shong YK (2005). 18F-fluorodeoxyglucose uptake in thyroid from positron emission tomogram (PET) for evaluation in cancer patients: high prevalence of malignancy in thyroid PET incidentaloma. Laryngoscope.

[6] Bleeker-Rovers CP, Vos FJ, Mudde AH (2007). A prospective multi-centre study of the value of FDG-PET as part of a structured diagnostic protocol in patients with fever of unknown origin. Eur J Nucl Med Mol Imaging.

[7] (2022). MedCalc Software Ltd. Diagnostic Test Evaluation Calculator (Version 20.100). https://www.medcalc.org/calc/diagnostic_test.php.

[8] Schönau V, Vogel K, Englbrecht M (2018). The value of 18F-FDG-PET/CT in identifying the cause of fever of unknown origin (FUO) and inflammation of unknown origin (IUO): data from a prospective study. Ann Rheum Dis.

[9] Kouijzer IJ, Mulders-Manders CM, Bleeker-Rovers CP, Oyen WJ (2018). Fever of unknown origin: the value of FDG-PET/CT. Semin Nucl Med.

[10] Vaidyanathan S, Patel CN, Scarsbrook AF, Chowdhury FU (2015). FDG PET/CT in infection and inflammation--current and emerging clinical applications. Clin Radiol.

[11] Hung BT, Wang PW, Su YJ, Huang WC, Chang YH, Huang SH, Chang CC (2017). The efficacy of 18F-FDG PET/CT and 67Ga SPECT/CT in diagnosing fever of unknown origin. Int J Infect Dis.

[12] Pereira AM, Husmann L, Sah BR, Battegay E, Franzen D (2016). Determinants of diagnostic performance of 18F-FDG PET/CT in patients with fever of unknown origin. Nucl Med Commun.

[13] Besson FL, Chaumet-Riffaud P, Playe M (2016). Contribution of (18)F-FDG PET in the diagnostic assessment of fever of unknown origin (FUO): a stratification-based meta-analysis. Eur J Nucl Med Mol Imaging.

[14] Weitzer F, Nazerani Hooshmand T, Pernthaler B, Sorantin E, Aigner RM (2022). Diagnostic value of F-18 FDG PET/CT in fever or inflammation of unknown origin in a large single-center retrospective study. Sci Rep.

[15] Holubar J, Broner J, Arnaud E (2022). Diagnostic performance of 18 F-FDG-PET/CT in inflammation of unknown origin: a clinical series of 317 patients. J Intern Med.

[16] Georga S, Exadaktylou P, Petrou I (2020). Diagnostic value of 18F-FDG-PET/CT in patients with FUO. J Clin Med.

[17] Wang Q, Li YM, Li Y (2019). 18F-FDGPET/CT in fever of unknown origin and inflammation of unknown origin: a Chinese multi-center study. Eur J Nucl Med Mol Imaging.

[18] Chen JC, Wang Q, Li Y (2022). Current situation and cost-effectiveness of 18F-FDG PET/CT for the diagnosis of fever of unknown origin and inflammation of unknown origin: a single-center, large-sample study from China. Eur J Radiol.

[19] Singh N, Kumar R, Malhotra A, Bhalla AS, Kumar U, Sood R (2015). Diagnostic utility of fluorodeoxyglucose positron emission tomography/computed tomography in pyrexia of unknown origin. Indian J Nucl Med.

[20] Crouzet J, Boudousq V, Lechiche C (2012). Place of (18)F-FDG-PET with computed tomography in the diagnostic algorithm of patients with fever of unknown origin. Eur J Clin Microbiol Infect Dis.

[21] Lee YH, Choi SJ, Ji JD, Song GG (2016). Diagnostic accuracy of 18F-FDG PET or PET/CT for large vessel vasculitis: a meta-analysis. Z Rheumatol.

[22] Soussan M, Nicolas P, Schramm C, Katsahian S, Pop G, Fain O, Mekinian A (2015). Management of large-vessel vasculitis with FDG-PET: a systematic literature review and meta-analysis. Medicine (Baltimore).

[23] Mason JC (2010). Takayasu arteritis--advances in diagnosis and management. Nat Rev Rheumatol.

[24] Nazareth R, Mason JC (2011). Takayasu arteritis: severe consequences of delayed diagnosis. QJM.

[25] Sharma BK, Jain S, Suri S, Numano F (1996). Diagnostic criteria for Takayasu arteritis. Int J Cardiol.

[26] Craven A, Robson J, Ponte C (2013). ACR/EULAR-endorsed study to develop Diagnostic and Classification Criteria for Vasculitis (DCVAS). Clin Exp Nephrol.

[27] Luqmani RA, Suppiah R, Grayson PC, Merkel PA, Watts R (2011). Nomenclature and classification of vasculitis - update on the ACR/EULAR diagnosis and classification of vasculitis study (DCVAS). Clin Exp Immunol.

[28] Johnston SL, Lock RJ, Gompels MM (2002). Takayasu arteritis: a review. J Clin Pathol.

[29] Arnow PM, Flaherty JP (1997). Fever of unknown origin. Lancet.

